# Understanding the unique and common perspectives of partners engaged in knowledge mobilization activities within pediatric pain management: a mixed methods study

**DOI:** 10.1186/s12913-024-10782-x

**Published:** 2024-03-14

**Authors:** Nicole E. MacKenzie, Christine T. Chambers, Christine E. Cassidy, Penny V. Corkum, Meghan E. McGrady, Jennifer A. Parker, Kathryn A. Birnie

**Affiliations:** 1 Department of Psychology and Neuroscience, Dalhousie University, Life Sciences Centre, 1355 Oxford Street, Halifax, NS B3H4J1 Canada; 2 Centre for Pediatric Pain Research, IWK Health, Halifax, NS Canada; 3 Department of Pediatrics, Dalhousie University, Halifax, NS Canada; 4 Solutions for Kids in Pain, Halifax, NS Canada; 5 School of Nursing, Dalhousie University, Halifax, NS Canada; 6 Department of Psychiatry, Dalhousie University, Halifax, NS Canada; 7 Department of Pediatrics, IWK Health, Halifax, NS Canada; 8 Division of Behavioral Medicine and Clinical Psychology, Cincinnati Children’s Hospital Medical Center, Cincinnati, OH USA; 9 Department of Pediatrics, University of Cincinnati College of Medicine, Cincinnati, OH USA; 10 Department of Anesthesiology, Perioperative, and Pain Medicine, and Community Health Sciences, University of Calgary, Calgary, AB Canada

**Keywords:** Knowledge mobilization, Partnership, Mixed-methods, Child health, Pediatric pain

## Abstract

**Background:**

Knowledge mobilization (KM) is essential to close the longstanding evidence to practice gap in pediatric pain management. Engaging various partners (i.e., those with expertise in a given topic area) in KM is best practice; however, little is known about how different partners engage and collaborate on KM activities. This mixed-methods study aimed to understand what different KM partner groups (i.e., health professionals, researchers, and patient/caregiver partners) perceive as supporting KM activities within pediatric pain management.

**Methods:**

This study used a convergent mixed-methods design. Ten partners from each of the three groups participated in interviews informed by the Consolidated Framework for Implementation Research, where they discussed what impacted KM activities within pediatric pain. Participants then rated and ranked select factors discussed in the interview. Transcripts were analyzed within each group using reflexive thematic analysis. Group-specific themes were then triangulated to identify convergence and divergence among groups. A matrix analysis was then conducted to generate meta-themes to describe overarching concepts. Quantitative data were analyzed using descriptive statistics.

**Results:**

Unique themes were developed within each partner group and further analysis generated four meta-themes: (1) team dynamics; (2) role of leadership; (3) policy influence; (4) social influence. There was full agreement among groups on the meaning of team dynamics. While there was partial agreement on the role of leadership, groups differed on who they described as taking on leadership positions. There was also partial agreement on policy influence, where health professionals and researchers described different institutions as being responsible for providing funding support. Finally, there was partial agreement on social influence, where the role of networks was seen as serving distinct purposes to support KM. Quantitative analyses indicated that partner groups shared similar priorities (e.g., team relationships, communication quality) when it came to supporting KM in pediatric pain.

**Conclusions:**

While partners share many needs in common, there is also nuance in how they wish to be engaged in KM activities as well as the contexts in which they work. Strategies must be introduced to address these nuances to promote effective engagement in KM to increase the impact of evidence in pediatric pain.

**Supplementary Information:**

The online version contains supplementary material available at 10.1186/s12913-024-10782-x.

## Background

Pediatric pain, defined as pain of any cause or duration in children from birth to 18 years of age, is a significant childhood health issue, and has immediate and long-term consequences that are physical (e.g., increased sensitivity to pain), psychological (e.g., development of mood and anxiety disorders, avoidance of medical care, etc.), and social (e.g., decreased school attendance, decreased social connectivity) [1–4]. Despite the availability of evidence-based pain management strategies, as well as calls to implement evidence into practice in the field of pain, knowledge of and access to evidence remain primary barriers among knowledge users in pediatric pain, such as health professionals, decision makers, and patients/caregivers [5, 6]. Knowledge mobilization (KM) activities are critical to close this knowledge-to-action gap through dissemination and implementation of evidence. KM can be described as activities that spread and support the use of evidence in practice, including dissemination, implementation, synthesis, and exchange [7]. Specific activities in pediatric pain have included caregiver-oriented videos (e.g., It Doesn’t Have to Hurt, Be Sweet to Babies) [8, 9], pain toolkits for caregivers and health professionals [10, 11], a pain curriculum for health professionals [12], and most recently, Solutions for Kids in Pain, a national knowledge mobilization centre for pediatric pain [13]. Best practice in KM includes developing partnerships, or collaborations between individuals or groups with relevant expertise and knowledge [14, 15]. Partnerships can be formed between a variety of knowledge users, who are impacted by, or are interested in, the outcomes of a KM activity [16, 17], and knowledge producers who generate knowledge. Partners exist within partnerships, and can be defined as individuals with unique skills and expertise who collaborate on KM initiatives [18]. Knowledge users and knowledge producers can therefore be considered “partners” in KM activities. In the context of pediatric pain, key partners include researchers, health professionals (e.g., nurses, psychologists, physicians, etc.), and patient/caregiver partners. Overall, to promote the use and impact of evidence in practice, multi-partner engagement in, and co-production of, KM initiatives are essential [19, 20].

Despite the known value of engaging partners in KM activities, differences among key partner groups (i.e., health professionals, researchers, and patient/caregivers), as it relates to their priorities within, approaches to, and perceptions of successful KM are not well understood. Understanding why different partners choose to engage in KM activities, and how they wish to engage, are critical to support effective partnership, yet key gaps remain in this area, including the need to identify strategies for engaging partners in KM activities, support team-based interactions, understand unique and/or shared priorities when engaged in KM, and manage partner expectations [21–24]. Relatedly, identifying what different partners view as most important to supporting their engagement in KM processes can highlight opportunities to increase engagement in KM and improve the translation of evidence into practice.

The Consolidated Framework for Implementation Research (CFIR) can be used to explore partner perspectives on what is most impactful when it comes to supporting KM activities [25]. The CFIR can facilitate the structured exploration of partners’ unique perspectives on what impacts KM activities in the context of pediatric pain and to what extent these concepts are valued. The CFIR can be used to understand the context of partner engagement in KM processes within pediatric pain, generate new hypotheses, and inform implementation strategies [25, 26].

The purpose of this mixed-methods study was to understand what different partner groups (i.e., health professionals, researchers, and patient/caregiver partners) perceive as being most impactful and important in supporting KM activities in pediatric pain. This included the exploration of both positive and negative factors that impact KM activities. Gaining this understanding can improve the mobilization of evidence by informing tailored KM strategies relevant to the pediatric pain context.

## Methods

### Study design

This study utilized a convergent mixed-methods design, an approach that compares qualitative and quantitative data for a comprehensive understanding of a concept [27]. Use of a mixed-methods design is recommended for implementation research in health service delivery contexts because of the detail and elaboration on these processes facilitated by this design [28]. The qualitative component of the study was informed by a qualitative description orientation, whereby participant experiences are described and summarised to understand factors related to health care systems and those who work within them [29]. The Consolidated Criteria for Reporting Qualitative Research (COREQ) guidelines were adhered to for reporting of this study (see Additional File 1 – COREQ Checklist).

### Participants

**Fig. 1:**
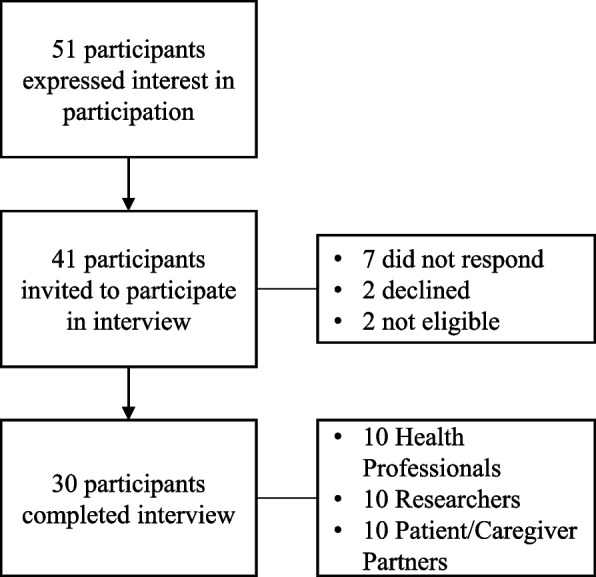
Recruitment flowchart

Participants were English-speaking health professionals (e.g., psychologists, physiotherapists, nurses, etc.), researchers (i.e., trainees, early-career to senior), and patients/caregivers with experience participating in at least one KM activity related to pediatric pain (e.g., educational material development, advisory board participation, resource development, etc.). Participants were recruited using convenience sampling via social media, e-newsletters, listserv messages, web pages, and emails to partner organizations (e.g., chronic pain clinics, etc.). Participation was open to participants within and outside of Canada. Of those who expressed interest in participation, maximum variation sampling was used to capture broad representation of experiences within each partner group [30]. A total of 51 individuals expressed interest in participation, 41 were invited to participate, and 30 ultimately participated, with 10 participants in each partner group (see Fig. 1—Recruitment flow chart). The sample size was selected a priori based on recommendations considering this type of research question and the analysis conducted [16, 31, 32].

### Measures

#### Interview guide

The semi-structured interview guide consisted of twelve open-ended questions pertaining to partners’ experiences participating in KM activities within pediatric pain, and addressed what partners perceived as impacting KM processes and outcomes (see Additional File 2 – Interview Guide). The interview guide was informed by the original version of the CFIR [33] (i.e., 1–2 questions related to each domain) and by an earlier survey on partner needs when accessing and applying evidence on pediatric pain management [5]. Previously published interview guides related to KM were also consulted to inform length and style of the questions [34, 35]. Following the development of the interview guide, it was reviewed by an external panel consisting of one representative from each partner group. The panel provided feedback on the content, relevance, and clarity of the questions, and the guide was adjusted accordingly.

#### Factor rating task

The two-question factor rating task presented participants with a pre-determined list of 10 factors derived from the CFIR that were included in the interview guide. Participants first rated factors in terms of their importance to successful KM initiatives in pediatric pain, using a Likert scale with the options “very important”, “somewhat important”, and “not important”. Participants then ranked their top three factors in terms of importance from the same list. A previously published rating task was consulted to inform design [36].

### Procedure

This study was approved by the IWK Health Research Ethics Board (REB# 1027459), and participants provided informed consent online. Prior to beginning the interview, participants completed an online demographics form and had access to list of key definitions to clarify the content that would be covered in the study (Additional File 3 – Key Definitions Handout). The individual semi-structured interviews were conducted virtually via video conferencing software (i.e., Zoom), an effective method to facilitate interviews in qualitative research [37, 38]. Prior to commencing interviews, the interviewer and interviewee briefly discussed the purpose of the study and answered questions regarding procedures. One cisgender woman, graduate-level interviewer (NEM) with experience in interviewing and qualitative research conducted the interviews, using the same interview guide with all partner groups. Field notes were recorded after each interview and reviewed to identify and explore relevant topics in subsequent interviews [39]. Interviews were audio recorded and transcribed verbatim. Transcripts were not reviewed by participants. Interview lengths ranged from 47 to 86 min (61.5 min on average). Directly following the interview, participants completed the factor rating task using online survey software (Qualtrics, 2020) [40]. Participants who completed all components received a $25 CAD online gift card.

### Data analysis

Data analysis occurred in two phases. The qualitative analysis was conducted first to avoid the potential for the quantitative data to influence the interpretation of the qualitative data. Upon completion of the qualitative analysis, the quantitative data was analyzed.

#### Interview

Following the completion of all interviews, data was analyzed using thematic analysis with a combined inductive and deductive approach, combining the characteristics of reflexive analysis with a structured data analysis approach [41]. This approach is appropriate when a framework is used to inform a study but data emerges beyond the scope of the framework [16]. Thus, while the CFIR was inherent within the interview content and represented the deductive component of the approach, it did not explicitly guide the analysis, though it was referenced in the interpretation of the findings. The analytic process was informed by the phases described by Braun and Clarke [41], beginning with data familiarization, development of codes, and assigning data to codes using line-by-line coding (conducted using NVivo data management software) [42]. Theme generation was then conducted by grouping initial codes into broader categories, refining and defining the themes, and then naming the themes. This process was conducted for each partner group and was led by the first author (NEM). Code review, categorization, and theme development were reviewed and discussed with another investigator (KAB) with experience in qualitative methods, as well as clinical, research, and KM expertise in pediatric pain. This process allowed for triangulation of results and maintained rigour in the analytic process. The patient/caregiver partner results were additionally reviewed and discussed with an external caregiver partner to offer perspective on any assumptions made during interpretation.

A descriptive matrix analysis was also conducted to identify themes that co-occurred among the partner groups [43]. Farmer and colleagues’ triangulation protocol informed the sorting and convergence coding process [44]. First, like-themes were sorted and a “meta-theme” (i.e., organizing concept) was generated. Then, the themes within the meta-theme were compared to explore the extent to which the themes agreed or disagreed on the core concept of the meta-theme. Agreement was defined as all themes from each group related to the same core meaning and the same context. Partial agreement was defined as themes that related to the same core concept but diverged in the examples or contexts raised. Dissonance was defined as themes within groups that differed in their meaning and context. Silence was defined as the presence of a theme within one or two groups that did not arise within another. The matrix and agreement ratings were generated by NEM and reviewed and discussed with KAB.

#### Demographics and factor rating task

Demographic data were analyzed by partner group using descriptive statistics and frequency counts. Factor rating data was analyzed between groups using frequency counts to determine overall ratings of importance for individual factors. Factor ranking data was also analyzed between groups using frequency counts to examine the prioritization of the factors.

#### Mixed methods integration

The qualitative and quantitative data were integrated following the steps for convergent designs [27]. Following the respective analysis of the qualitative and quantitative data, the results were compared to explore how the findings converged, diverged, or expanded on each other [27]. The data integration interpretation was incorporated within the overall discussion of the results.

## Results

### Participant characteristics

There were 30 participants in total, with 10 participants in each partner group (i.e., health professionals, researchers, and patient/caregivers). Participants predominantly identified as cisgender women of ethnic and/or cultural European origins (see Table 1 for all demographics). Health professionals had an average of 13.70 years of experience engaging in KM (*SD* = 6.98), had been predominantly engaged as a staff member or collaborator on a KM activity (e.g., patient education resources, advisory board participation), and mostly had experience with either implementation and/or dissemination (see Table 2). Researchers had an average of 12.30 years of experience engaging in KM (*SD* = 10.95), had predominantly led a KM initiative (e.g., infographics, clinical practice guidelines, policy guidelines, etc.), and had more experience with dissemination activities. Finally, patient/caregiver partners had an average of 9.60 years of experience engaging in KM activities (*SD* = 8.36), had predominantly been engaged as a partner on a KM initiative (e.g., social media campaigns, decision aid development, etc.), and had more experience with dissemination.

**Table 1:** Demographics

	Partner Group			
	Health Professional *n* (%)	Researcher *n* (%)	Patient/Caregiver Partner *n* (%)	
Gender Identity				
Cisgender Woman	8 (80.0)	9 (90.0)		10 (100.0)
Cisgender Man	2 (20.0)	1 (10.0)		0 (0.0)
Age				
18–29	0 (0.0)	0 (0.0)		5 (50.0)
30–39	3 (30.0)	6 (60.0)		2 (20.0)
40–49	4 (40.0)	3 (30.0)		2 (20.0)
50–59	2 (20.0)	1 (10.0)		1 (10.0)
60 or greater	1 (10.0)	0 (0.0)		0 (0.0)
Ethnicity (origins)				
Aboriginal NA	0 (0.0)	0 (0.0)		0 (0.0)
North American	1 (10.0)	0 (0.0)		1 (10.0)
European	9 (90.0)	9 (90.0)		7 (70.0)
Caribbean	0 (0.0)	0 (0.0)		1 (10.0)
Latin, Central, SA	0 (0.0)	0 (0.0)		0 (0.0)
African	0 (0.0)	0 (0.0)		0 (0.0)
Asian	0 (0.0)	0 (0.0)		3 (30.0)
Oceania	0 (0.0)	1 (10.0)		0 (0.0)
Country				
Canada	9 (90.0)	4 (40.0)		10 (100.0)
United States	1 (10.0)	1 (10.0)		0 (0.0)
Australia	0 (0.0)	3 (30.0)		0 (0.0)
Spain	0 (0.0)	1 (10.0)		0 (0.0)
UK & Northern Ireland	0 (0.0)	1 (10.0)		0 (0.0)
Geographic Region				
British Columbia	0 (0.0)	0 (0.0)		1 (10.0)
Alberta	3 (30.0)	1 (10.0)		0 (0.0)
Saskatchewan	1 (10.0)	0 (0.0)		0 (0.0)
Ontario	4 (40.0)	2 (20.0)		9 (90.0)
Quebec	0 (0.0)	1 (10.0)		0 (0.0)
Nova Scotia	1 (10.0)	0 (0.0)		0 (0.0)
International	1 (10.0)	6 (60.0)		0 (0.0)

*N* = 30, *n* = 10 in each partner group

**Table 2:** Knowledge mobilization roles held and experiences

	Partner Group		
	Health Professional *n* (%)	Researcher *n* (%)	Patient/Caregiver Partner *n* (%)
Duration of KM experience (years)			
1–5 years	1 (10.0)	3 (30.0)	4 (40.0)
6–10 years	2 (20.0)	3 (30.0)	2 (20.0)
11–15 years	3 (30.0)	2 (20.0)	3 (30.0)
16 + years	4 (40.0)	2 (20.0)	1 (10.0)
Roles held			
Consultant	3 (30.0)	3 (30.0)	6 (60.0)
Partner	3 (30.0)	1 (10.0)	9 (90.0)
Collaborator	7 (70.0)	8 (80.0)	6 (60.0)
Project Leader	5 (50.0)	10 (100.0)	2 (20.0)
Decision Maker	5 (50.0)	1 (10.0)	1 (10.0)
Staff Member	9 (90.0)	3 (30.0)	2 (20.0)
Identity as other partner type			
Health Professional	0 (0.0)	5 (50.0)	1 (10.0)
Researcher	2 (20.0)	1 (10.0)	3 (30.0)
Patient/Caregiver/Family	0 (0.0)	4 (40.0)	1 (10.0)
Clinician-Scientist	1 (10.0)	0 (0.0)	0 (0.0)
Other	0 (0.0)	1 (10.0)	2 (20.0)
Context of role			
Hospital/Clinic	10 (100.0)	7 (70.0)	5 (50.0)
University/Academic	3 (30.0)	9 (90.0)	9 (90.0)
Private Industry	0 (0.0)	0 (0.0)	1 (10.0)
Government	1 (10.0)	1 (10.0)	2 (20.0)
Other	0 (0.0)	2 (20.0)	1 (10.0)
Number of teams engaged with			
1 team	4 (40.0)	2 (20.0)	1 (10.0)
2–4 teams	4 (40.0)	5 (50.0)	4 (40.0)
5–10 teams	2 (20.0)	3 (30.0)	5 (50.0)
Experience type			
More implementation	3 (30.0)	1 (10.0)	1 (10.0)
More dissemination	3 (30.0)	5 (50.0)	5 (50.0)
About equal of both	2 (20.0)	2 (20.0)	3 (30.0)
Did not answer	2 (20.0)	2 (20.0)	1 (10.0)
Type of KM Activity			
Advisory board participation	3 (30.0)	0 (0.0)	3 (30.0)
Institution accreditation standard	1 (10.0)	0 (0.0)	0 (0.0)
Clinical practice guideline initiative	2 (20.0)	1 (10.0)	0 (0.0)
Evidence consultations	1 (10.0)	0 (0.0)	0 (0.0)
Provision of educational workshops/presentations	5 (50.0)	1 (10.0)	0 (0.0)
Infographic development	1 (10.0)	2 (20.0)	0 (0.0)
Patient/parent education resources	6 (60.0)	6 (60.0)	2 (20.0)
Policy guideline/change development	2 (20.0)	2 (20.0)	1 (10.0)
App development	0 (0.0)	2 (20.0)	2 (20.0)
Blog posts	0 (0.0)	1 (10.0)	1 (10.0)
Decision aid development	0 (0.0)	0 (0.0)	1 (10.0)
Patient organization	0 (0.0)	0 (0.0)	1 (10.0)
Co-presentation	0 (0.0)	0 (0.0)	3 (30.0)
Social media outreach	0 (0.0)	0 (0.0)	3 (30.0)
Website development	0 (0.0)	0 (0.0)	1 (10.0)
Media Pieces	0 (0.0)	1 (10.0)	0 (0.0)
Presentations to general public	0 (0.0)	1 (10.0)	0 (0.0)

*N* = 30; *n* = 10 in each partner group

### Qualitative results

#### Health professionals

Four themes were generated for health professionals. Theme are discussed below, with theme components italicized. Illustrative quotations for each theme appear in Table 3.

**Table 3:** Health professional perspectives on what supports knowledge mobilization

Theme	Theme Component	Quotation
THEME 1: Leaders champion and motivate teams	Clear identification and recognition of who was leader	*One thing that I found really helpful is having one person being the lead. That way there's inputs from multiple people, but it's recognized that that one person is kind of responsible for moving things along.* [9411]
	Leaders as champions who bring enthusiasm and skill to their team	*Our nurse practitioner really was very enthusiastic and involved in a broad variety of initiatives across the hospital with both acute pain and chronic pain. And so I think some of her enthusiasm and her skill set really rubbed off on the rest of us as well.* [4304]
	Providing support to all team members	*I think there should be some real-time feedback…sending out to staff, saying, “Okay, you know, last month we had five percent of kids get topical with their needle pokes. And this month we got 20 percent. Good job. We're aiming for 50.”…So then they're like, “Oh, okay, wow, this is making a difference.”* [7949]
THEME 2: Context matters	Taking a “bottom up” or partner-informed approach to KM activities	*…If you have emerging leaders that can feel like they're a part of a change and really have those ties to it, and can be the ones recognizing the barriers, but then coming up with strategies to address them, too. …I think we’d see more implementation and we would see better uptake if that's the case.* [6122]
	Identify and address the practical needs of KM partners	*That's a classic for staff nurses. Like they want them to contribute to a thing, and it's on their own time. Or during their 12-h shift that they have to stay after or go on their lunch. So that's another way to value them, is, you know, just ensuring that that those kinds of things are there—renumeration, you know, that you're actually given a time where you're not also working.* [7949]
	Gaining insight from multiple team members	*…It's feeling comfortable being able to present those ideas… And then from there, we'll make decisions not based on necessarily hierarchy or position, but whether it will best suit the needs of the kids.* [9411]
THEME 3: Investment in relationships with common values	Developing and leveraging relationships	*Everything starts off with relationships, right? And so I think the more relationships that you have that are strong, the more likely you are to be able to get somebody on side and be willing to sway their network of people that exist for them, or to sway their entire organization.* [0325]
	Investing in existing relationships to develop new connections	*Stakeholder investment is certainly important. So you know, when we launched the survey… we made sure to have our nurse manager is on our committee because she's well-connected within the clinical leader groups at the [hospital].* [6367]
	Establishing shared values among partners	*…We need to start off by ensuring that we've got similar value sets. Meaning that, you know, are we going to prioritize telling every inch of the truth, which can sometimes be hurtful or harmful? Or are we going to prioritize the well-being of the child and family? … I think it's really important that the team all be on the same page about what the game plan is going to be. And I think that's based on our values.* [0325]
	Respect for partners’ unique perspectives	*Like there has to be that kind of mutual respect and understanding of each person's clinical role, kind of what they bring to the table. Because each of us is going to have a different approach to how we're dealing with pain, and kind of how we're dealing with anxiety and education.* [8955]
THEME 4: Knowledge mobilization initiatives need decision maker support	Funding and decision maker support for implementing initiatives	*For example, it would be my goal to, as an organization…[to] receive ChildKind certification. That we would be able to kind of show the community that we have made a commitment to children's pain, and these are the ways.* [6122]
	Funding and policy support determines the feasibility of a KM initiative	*The pediatric working group with the Ministry of Health that I was involved in, it was totally guided by the current minister who took chronic pain on as a project. … So the only reason that got off the ground is because that current minister said, “Make this so.” …Policy priorities, funding priorities. That led to funding of our program. That helped us implement evidence-based care that we couldn't before.* [7949]

##### Theme one: leaders champion and motivate teams

Leaders were described as those who recognized a need and guided teams through developing and implementing KM activities (e.g., nurse practitioner leading implementation of educational programming), and were seen a key source of support for KM initiatives. When there was *clear identification and recognition of the leader*, team members responsibilities were understood, as was who to turn to regarding questions and direction. Additionally, leaders were seen as *champions who bring enthusiasm and skill to their team*, who in turn, were perceived as motivating their team members. These characteristics were especially important when the KM initiative was complex or required significant practice change. Finally, leaders were described as responsible for *providing support to all team members*, especially those apprehensive about change, by sharing progress updates and evidence of change to demonstrate an initiative’s impact.

##### Theme two: context matters

Consideration of the implementation context, described as the structure and values of both the team and the broader organization, was shared by most health professionals as being essential to successful KM activities. *Taking a “bottom up” or partner-informed approach to KM activities* was illustrated as a context-driven method to developing a KM initiative and was perceived as most successful when ideas emerged from within the team, earning buy-in and ensuring relevance. Another component was the need to *identify and address the practical needs of KM partners*. Many health professionals recognized the burden put on clinical staff on teams (e.g., making time for KM team meetings) and discussed the importance of addressing these practical needs to alleviate barriers to participation (e.g., providing clinical coverage). Finally, health professionals described the importance of *gaining insights from multiple team members* with knowledge of the pediatric pain context. This was seen as critical to comprehensively considering the context when planning the KM initiative.

##### Theme three: investment in relationships with common values

Health professionals identified relationships with other partners (e.g., other health professionals, patient/caregivers, researchers) as supporting KM initiatives in pediatric pain. *Developing and investing in relationships* with other partners was seen as a key to gaining traction and support for a KM initiative, as was *leveraging existing relationships* to support ongoing and future KM initiatives in the long-term. In order to achieve that support, *investment in existing relationships to develop new connections* was integral, as a relationship with one partner had the potential to facilitate connections with other who could support, and perhaps benefit from, the KM initiative. At the core of maintaining and leveraging well-established relationships was *establishing shared values among partners*. Health professionals described that common values and goals related to KM (e.g., valuing engagement in KM) supported the ease with which cohesive decisions could be reached when developing and implementing KM initiatives. A key value within achieving this was *respect for partners’ unique expertise* within pediatric pain, supported by consideration for preferred communication styles, as well as individuals’ priorities and capacity for engaging in KM initiatives, ultimately facilitating the integration of each partners’ unique perspective.

##### Theme four: knowledge mobilization initiatives need decision maker support

Health professionals desired to implement best practice standards to improve pain care for children; however, these practices were described as most successful when there was *funding and decision maker support for implementing initiatives*. Initiatives such as voluntary certifications (e.g., ChildKind) [45] were important to promote the implementation of best practices for pain management to address patient needs; however, such initiatives required support from funders to meet standards. Health professionals described that whether decision makers could provide *funding and policy support* often determined the feasibility of a KM initiative, irrespective of the value of a pediatric pain management initiative. When policy support and funding were granted, health professionals described the substantial ease with which implementation could happen.

#### Researchers

Five themes were generated for researchers. Theme are discussed below, with theme components italicized. Illustrative quotations for each theme appear in Table 4.

**Table 4:** Researcher perspectives on what supports knowledge mobilization

Theme	Theme Component	Quotation
THEME 1: The mixed value of knowledge mobilization in academic systems	Academic institutions may view KM as secondary to traditional academic outputs	*All the systems are set up in terms of numbers of papers… I can definitely say that that hampers a lot of people. Like if I say to you, “Oh, you should submit a thing for TED-Ed. It’ll only take a few hours,” a lot of people go, “Oh, but that's not going to lead to a paper. And so it's not worth my time.”* [0509]
	Funding agencies’ value of KM influences support available	*I don't think they understand implementation science because they all say the effectiveness has been proven, what are you doing? Like just do it. And like, yeah, but we need to implement it. … So, I’m not getting the funding.* [1276]
	External policies and incentives as motivators for researchers to partake in KM activities	*But that [funding agencies are] looking, from what I understand, more fondly upon work that can demonstrate it has impact rather than just, you know, occurring in research journals. But if it can be translated, it's like a necessary part now.* [8735]
THEME 2: A perceived need for greater knowledge of knowledge mobilization processes	Lacking an understanding of formal KM processes and theory	*I don't even know how to describe it…I often don't have the lingo necessarily and use the lingo that other people do.* [3261]
	Degree of clarity on KM processes influenced whether researchers felt they had the skills to engage in it	*… I thought I know nothing about knowledge translation. In fact, I did. Because I was a clinical nurse educator, I’d been doing these things for years in my own practice. But I felt I knew nothing. And in fact, when I learned about what [KM] is, I thought yeah, I do that, I've been doing that, I've done that.* [3363]
	Lacking certainty around when to engage in KM	*…To translate that knowledge to the people with lived experience of pain or clinicians [who] can access it, sometimes I worry… do we have enough information on the research side of things to show that it has a benefit and doesn't have a harm before we do that? And so my hesitation in engaging more in the knowledge translation is that I don't know if we have those answers yet to do that in the space that I'm in.* [8735]
	Knowledge of KM provides guidance and confidence	*You sort of have this method that is ready to go, tested, developed oftentimes by brilliant scientists. You sort of, again, buy in. You can trust that kind of branding. And then in that way, supports the implementation of your project.* [0903]
THEME 3: Leveraging colleagues’ knowledge mobilization visibility to support implementation	Colleagues’ successful KM projects bring credibility and acceptability to implementation initiatives	*…To have been able to come in and say we're part of this unit that has been developed by [name], whatever we say kind of goes. It's very weird and very powerful and very cool.* [0460]
	Branding of an initiative communicates credibility	*…There's a bit of a branding thing to leverage the notoriety, the strength of these organizations to give your implementation more credibility… [it] helps with uptake because people realize that you're on to something and you've partnered with these groups that are doing great things.* [0903]
	Uneasiness in developing branding of KM initiatives	*…I think people put a face on [KM]. And they don't have to do that, right. They can just do the knowledge translation work. But sometimes they put their face on it, and put a flag in, and they're like, “It's my work.” And I'm not always sure, I’m like is that what we should be doing? Shouldn't we just be translating it rather than being like it’s my knowledge translation work?* [8735]
THEME 4: Strong project teams emerge from collaborators with diverse expertise	Inclusion of diverse voices can ensure the relevance and impact of a KM initiative	*We're always engaging patient partners, family member partners, other people with lived experience, clinicians, and then, of course, researchers as well in the work that we do. So I think that at the end of the day that they're kind of more ready for dissemination.* [0903]
	Presence of team members with practical know-how of how to share evidence	*I can give you the information that needs to be in there, and somebody else needs to put their magic wand on it to make it look beautiful and attractive, and people will want to read it.* [1276]
	Necessity for connection and understanding between team members	*…One thing I'm doing constantly is talk as much as you can with the clinicians…These type of folks who can understand both things really well become critical, you know, because they understand the complexity of the research that is being carried out, and to which extent we can actually conclude something…* [9192]
	Connections with colleagues and changemakers	*…Have a few friends in those domains where you can actually ask important questions. Like, “Hey, I don't get this. Can this be helpful for me? I'm trying to elucidate this question. Can this method help me or not?” You know, be able to have people that have that knowledge and that you can trust to have a conversation, right.”* [9192]
THEME 5: Collaborative leadership is idealized	Collaborative leaders leverage the skills and strengths of team members	*I think the co-design work that I do as a researcher is emblematic of my leadership style in general. Which is really to lead from the middle and sort of organize a team around me, recognizing everybody's strengths, and leveraging those to do work. And I think that's quite an effective way to get the buy-in on the units to do this type of implementation work.* [0903]
	Mutual respect and a shared vision of the KM initiative	*I mean I think having shared goals and a shared vision for engaging in the implementation process, for hearing other people's perspectives, and not coming in and saying as the expert and thinking that this is the way that you need to change clinical practice. Versus hearing from the system and taking time to understand the system, understand the needs of the system.* [5136]
	Leaders collaboratively determine roles, expectations, and boundaries	*I'm personally just learning to try and have those conversations as soon as possible and just get all the roles really, really clear…Clarify everything right upfront, and like, this is how I'm going to attend meetings, and this is how often I can look at drafts or whatever.* [0509]
	Acknowledging power hierarchies is essential to manage them	*I think if there is a power imbalance, but it's either pretended not to be there, and it's not sort of clear about what people's roles are, that becomes a challenge, right. And people tend to butt heads, I think, when they thought they had one type of relationship and then it switches.* [3261]

##### Theme one: the mixed value of knowledge mobilization in academic systems

Researchers described that the value placed on KM activities by funders or academic institutions influenced the extent to which some researchers engaged in KM. Some researchers described that *academic institutions may view KM as secondary to traditional academic outputs* (e.g., publications, grants). They described that the extent to which their institution valued KM influenced how easily time spent engaging in KM could be justified. Researchers also described that *funding agencies’ value of KM may also influence support*. Whether funders valued or even understood KM influenced the degree to which they were supported financially. Others described *external policies and incentives as motivators* for researchers to partake in KM activities, as when KM activities were required by funding agencies, researchers sometimes described reconsidering the value of partaking in them.

##### Theme two: a perceived need for greater knowledge of knowledge mobilization processes

 Knowledge of KM processes was an important component of confidence when engaging in KM. Many researchers felt they *lacked an understanding of formal KM processes and theory*, and seemed to regard it as an esoteric construct, which impacted confidence. Jargon (e.g., KM-specific terminology, theory, frameworks) further increased inaccessibility and challenges understanding what KM was or how to engage in it. A lack of clarity on KM processes and theory influenced whether researchers felt they had the *skills to engage* in it. For some, it was not until they learned about the activities and processes that constitute KM that they recognized their work as KM. Other researchers expressed a *lack of certainty around when to engage in KM*. Hesitancy to engage in KM within pediatric pain was often brought on by ethical concerns around whether sufficient evidence was available to translate confidently, and whether enough was known about how to do KM. *Knowledge provided guidance and confidence* when available, however. Researchers who had knowledge about theories, models, and frameworks, and how they could support KM, described greater confidence through the guidance offered by these concepts.

##### Theme three: leveraging colleagues’ knowledge mobilization visibility to support implementation

Researchers described that *colleagues’ successful and notable KM projects often brought credibility and acceptability* that was subsequently respected by collaborators and earned their buy-in (e.g., a KM resource on needle pain management shared by Solutions for Kids in Pain). Researchers described other partners as more willing to engage when they were implementing a recognizable, branded KM initiative, where *branding communicated credibility* through familiarity and trust in those who created the initiative. Others researchers, however, described an *uneasiness of developing branding* to promote and gain traction with their initiative. While branding of KM initiatives conflicted with some researchers’ values, the influence of branding and recognizability appeared to be valuable to harness in support of KM in pediatric pain.

##### Theme four: strong project teams emerge from collaborators with diverse expertise

Collaborative engagement was described as *including diverse voices with relevant expertise* for the project, including diversity in professional background and lived experience. This was integral to ensure the relevance and impact of a KM initiative through inclusion of those who would potentially be impacted by the KM initiative. Researchers described diversity in expertise as including *team members with practical know-how* related to the evidence, the KM process, as well as other relevant skills (e.g., graphic design, communications, etc.) to tailor information for a pediatric pain audience. *Connection and understanding between team members* were critical foundations to ensure diversity was maximally harnessed, with consistent communication being key to understanding the perspectives of all team members and the complexities within the KM context. In order to gain diversity in perspectives, *connections with colleagues and changemakers* in the field were critical. Researchers described networks as fundamental to seeking collaboration opportunities to access necessary expertise.

##### Theme five: collaborative leadership is idealized

Researchers valued and preferred collaborative team leaders who were able to *leverage the skills and strengths of team members* to develop and execute KM initiatives. Collaborative leadership encompassed power sharing with team members by incorporating their perspectives to the extent possible. Some researchers described the core of collaborative leadership as *mutual respect and a shared vision of the KM initiative*. Researchers discussed the importance of taking time to establish connections and trust as the foundation of a relationship, promoting productive interactions throughout a project. Practically, researchers idealized leaders who *collaboratively determined roles, expectations, and boundaries*, as mutual expectation setting was described as key to facilitating a positive team dynamic and achieving KM goals. Collaborative leadership also involved *acknowledging power hierarchies in order to manage them*. Power hierarchies led to tension and challenging communication within teams; however, when leaders acknowledged power differentials and opened lines of communication, researchers believed these differentials could be managed to ensure productive engagement in KM initiatives.

#### Patient/caregiver partners

Four themes were generated for patient/caregiver partners. Theme are discussed below, with theme components italicized. Illustrative quotations for each theme appear in Table 5.

**Table 5:** Patient/caregiver perspectives on what supports knowledge mobilization

Theme	Theme Component	Quotation
THEME 1: Value and trust stem from belongingness	Importance of facilitating belongingness and safety	*I find that like there are certain environments like where you're, again, valued as a whole person and you're like, “Hey, [participant], how are you doing today?” Just like even basic human connection moments. … I'm here for more than just sharing, you know, one or two sentences about my life.* [7827]
	Belongingness communicates the value of the patient/caregiver perspective	*I think I'm already coming to the table with “I'm not equal”. I think it's getting better, but… It's sort of like… everyone always says, “Oh, your voice is so valuable,” whatever. But are you really listening or are you just going to go on…* [0374]
	Specific actions to demonstrate a commitment to facilitating belongingness	*It doesn't make sense to have a whole team of paid team members, and then you invite a bunch of parent partners…and their expertise in that lived experience, and then not pay them. That does not help with softening edges of our hierarchy.* [1792]
THEME 2: Accessibility in patient/caregiver relationships is facilitated by leaders	Life experiences can impact one’s ability to participate	*I had to reflect on how [the resource] would have impacted me in terms of like my fear of [illness] number one as a child… And like that's a heavy topic, right. It's emotional. It's vulnerable.* [5736]
	Pain-related barriers can impact ability to participate in KM activities	*There's a big misconception that you're kind of flaky like when you live with a disability. And it's really just like you need flexibility. It's not to do with like your level of commitment. Because you can be totally committed to a project and like be passionate about it, but your health is a roadblock.* [7827]
	Structured opportunities and adaptations to participate in KM activities are important to create space for contributions	*And the person who was leading the group was really conscious of letting [patient partner] speak. Like giving her more floor time because she spoke slower, because it was a little bit harder to understand over Zoom, because her words were so wise.* [0374]
	Team leaders are viewed as responsible for identifying and enacting accommodations	*But [the leaders are] very mindful… Like they'll do all these check-ins…You know that if you have something to say, you can. Or you're constantly reminded that you don't have to talk about things. …It's reassuring. Because if you ever do feel like you want to maybe stop and backtrack and take a bit of a different path, you know that you can.* [9798]
THEME 3: Engaging patient/caregiver partners with relevant expertise	Value and respect for lived experience as expertise	*Patients have for a very long time been considered the bottom of the totem pole. And I think that's because our stories weren't valued. …It's not just about the science, it's about how it affects me as a person.* [3236]
	Including diversity of lived experience increases utility of KM activities	*…I think you also need a variety of expertise and lived experience… Just because my experience could be drastically different than the person sitting next to me… It's hard to make a collective when you only have kind of one perspective.* [2112]
	Necessity for greater representation and diversity of patient/caregivers as partners in KM activities to inspire others to participate	*I feel proud of myself when I'm that representation. Like I'm in that [output] for the project. And I'm like, look, it's somebody that looks like you. Like I promise you, you're not weird for doing this*. [7827]
	Expertise of the patient/caregiver partner must match the KM activity	*Maybe it's easy to pick the [partner] you already have a relationship with, but they don't really have… This parent isn't in [the illness] community… It will inform the research from the very beginning if that person has experience with that specifically. But it also helps at the very end when you're trying to share it with the broader or with perhaps a more niche group of people.* [1792]
THEME 4: Networks serve as communities	Networks of patient/caregiver partners spread evidence and resources, and facilitate connections between partners	*Informal networks can be incredibly helpful just for matching people with new projects. You know, if a researcher comes to us and asks us… “Oh, I don't know anybody. No one seems to know anybody that would be part of like a more marginalized group to participate on the research,” right. And we found… we work with like two people regularly.* [0015]
	There is expertise in navigating networks to effective share resources and knowledge	*And that's what I think the network is really all about. It's about whatever supports you have, that's great, that's fantastic, but what are you missing? And then how can we provide that for you, to you? Is it available, first of all? And if it isn’t, okay, what can be available, and then how do we get it, how do we deliver it?* [9798]
	Networks as a place of connection and trust	*…I have that network of people to talk to and be like what is appropriate compensation, what is, you know, the right way to navigate this? But if I didn't have that, and I was young and I didn't have that experience, I don't know how comfortable…Like I think I would have just kind of gone with whatever was happening.* [3236]

##### Theme one: value and trust stem from belongingness

Patient/caregiver partners described the value of humanity in interactions with team members in *facilitating belongingness and safety* in sharing perspectives. Taking time to connect as humans by expressing interest in the patient/caregiver partner’s life and well-being, as opposed to focusing exclusively on the pain related KM project, was described as building the foundation of relationships and trust, and subsequently facilitating openness to sharing. Instilling a sense of *belongingness communicated the value of patient/caregiver perspectives*, especially important when power differentials were perceived, as many patient partners felt unsure of the value of their contributions when working alongside partners who traditionally held power (e.g., health professionals, researchers). *Specific actions to demonstrate a commitment to facilitating belongingness* were a critical component of patient/caregiver engagement to support KM processes. Specifically, ongoing communication was described as signaling the value of the patient/caregiver perspective, with the absence of communication resulting in a feeling of exclusion. Compensation was also integral to communicating the equitable value of patient/caregiver partners’ contributions relative to other partners.

##### Theme two: accessibility in patient/caregiver relationships is facilitated by leaders 

Patient/caregiver partners described having *life circumstances and experiences that impacted their ability to participate* in KM activities related to pediatric pain, such as emotional readiness and consequences of sharing. The emotional toll of sharing lived experience impacted some patient/caregiver partners’ willingness to share, especially when trust had not yet been established. Patient/caregiver partners also discussed *pain-related barriers* that had interfered with their ability to complete tasks (e.g., difficulty recording notes due to arthritis-related pain), and described feeling judged for their impacted capacity. A specific adaptation raised by many patient/caregiver partners was providing *structured opportunities for participation*. Whether hampered by pain, or experiencing challenges making spontaneous contributions in groups settings with power hierarchies at play, designating opportunities to contribute during or after team interactions was described as increasing comfort and willingness to participate. It was ultimately *team leaders who were perceived as being those responsible for identifying and enacting these accommodations*. This included collaboratively planning for how patient/caregiver partners’ perspectives related to pediatric pain would be integrated into the KM initiative. When leaders took initiative to help patients/caregivers navigate partnership, many felt that they could contribute more effectively.

##### Theme three: engaging patient/caregiver partners with relevant expertise

Patient/caregiver partners discussed the importance of teams having *value and respect for lived experience* as expertise in its own right, a factor perceived to set the stage for the meaningful integrating of partner perspectives in decisions and KM outputs. The notion of engaging partners with relevant experience also included *diversity of lived experience*. The responsibility of representing all patient/caregiver voices could not fall to one individual, both due to the burden of being the single patient/caregiver partner, but also due to the limited scope of one’s own lived experience. Rather, the inclusion of multiple perspectives with lived experience with pain was regarded as increasing the general utility of KM outputs (e.g., social media campaigns for pain management, development of patient-facing pain management resources, etc.). Diversity in patient/caregiver partner representation was also discussed as having the potential to motivate others with lived experience to participate in KM initiatives. As such, *greater representation of diverse individuals* (e.g., physically, culturally, etc.) in KM was seen as an opportunity to engage more patient/caregiver partners, filling a critical need to include more heterogeneous pain experiences in KM initiatives. This was qualified with the need for balance, however, as patient/caregiver partners described the importance of partners having *relevant expertise to the KM activity*. A mismatch between a partner’s expertise and the KM initiative raised concerns around tokenism in partners who felt they were being included to simply “check a box”. Partners with relevant expertise were necessary to ensure meaningful engagement and relevant contributions.

##### Theme four: networks serve as communities

Networks of patient/caregiver partners in pediatric pain held a great deal of power to *spread evidence and resources, and facilitate connections between partners* to support KM initiatives. Networks were described as a place for patient/caregiver partners to share opportunities for partnership on KM initiatives in an effort to connect project teams with other patient/caregiver partners. Patient/caregiver partners also described their expertise in *navigating networks and sharing resources and knowledge* by tailoring their engagement approach with other partners. This was seen as leading to direct and impactful dissemination of KM initiatives. Access to networks was not privy to all and there was a sense of responsibility to maintain the safety of patient/caregiver networks in pediatric pain. Networks were described as *a place of connection and trust*, facilitated by privacy within these communities, where individuals could ask questions, seek advice or mentorship, or consult others. For many, this led to increased confidence and willingness to participate in KM related to pediatric pain.

#### Matrix analysis and triangulation

The matrix analysis and triangulation generated four meta-themes across the three partner groups (see Table 6 for detailed descriptions): (1) team dynamics (i.e., ideal team characteristics); (2) role of leadership (i.e., effective leadership components); (3) policy influence (i.e., the role of policy on the success of KM initiatives in pediatric pain); and (4) social influence (i.e., the role of social and network-related influences on KM initiatives in pediatric pain). There was agreement within the *team dynamics* meta-theme, and partial agreement within the *role of leadership*, *policy influence*, and *social influence* meta-themes; however, there were two group-specific themes (i.e., *a perceived need for greater knowledge of knowledge mobilization* and *context matters*) that did not converge with others, likely due to the specificity of the content to the respective partner group (see Table 6 for detailed descriptions).

**Table 6:** Matrix analysis and theme triangulation

Meta-Themes	Meta-Theme Description	Partner Group Themes			Interpretation
		Health Professionals	Researchers	Patient/Caregiver Partners	
Team Dynamics	This meta-theme captured themes related to productive team dynamics. Each group provided examples where the inclusion of team members with diverse perspectives, shared values, and strong relationships between them was key to team functioning and ensuring KM activities were relevant in the contexts they would be implemented	Investment in relationships with common values	Strong project teams emerge from collaboration with diverse expertise	Value and trust stem from belongingness Engaging patient/caregiver partners with relevant expertise	Agreement Having teams with diverse perspectives and strong relationships were consistent keys to KM success among all partners
Role of Leadership	This meta-theme captured themes that described effective leadership for KM teams. All partners agreed that leaders were responsible for structuring the KM process, ensuring progress, and supporting team members. Leaders were seen as core to the progress and outcomes of a KM initiative	Leaders champion and motivate teams	Desire to lead collaboratively	Accessibility in patient/caregiver relationships is facilitated by leaders	Partial Agreement Leaders were collectively seen as those responsible for planning, leading, and motivating; however, who was identified as leader differed among partners. Researchers described a desire to lead collaboratively and typically spoke from the perspective of a leader. Health professionals also tended to refer to themselves or colleagues as leaders. Patient/caregiver partners rarely referred to themselves as a leader, typically referencing another person (i.e., researcher or health professional)
Policy Influence	This meta-theme captured the role of policy on the success of KM initiatives in pediatric pain. Both researchers and health professionals described external influences on policy as impacting funding and KM opportunities for pediatric pain management. This included the role of support from academic institutions or policy makers at the ministerial/legislative level	Knowledge mobilization initiatives need decision maker support	Knowledge mobilization has mixed value as academic currency	Silence	Partial Agreement Researchers and health professionals both cited external influences as influencing resources for KM initiatives. Researchers described the impact of academic institutions’ value of KM on funding and weighting of KM activities when considering a researcher’s productivity. Health professionals spoke mainly about the impact that policy makers have on funding for KM activities within pediatric pain. This specific concept was less prevalent among patient/caregiver partners
Social Influence	This meta-theme captured the role of social and network-related influences on KM initiatives in pediatric pain. Health professionals, researchers, and patient/caregiver partners all cited social influences (e.g., influential nature of initiative, social network) as methods of getting resources and support for KM initiatives. Social connections had the potential to impact how KM activities were carried out among these groups	KM initiatives need DM support	Leveraging colleagues’ KM visibility to support implementation	Networks serve as communities	Partial Agreement Both the patient/caregiver partner and researcher groups raised the impact of social influences on support for spreading evidence and tapping into expertise within one’s network. Patient/caregiver partners’ discussion of social influences were unique in that they also discussed the role of networks as a social space where they could seek guidance related to participation in KM initiatives. Health professionals described networks as a place to learn about standards and activities used by other institutions, with the activity of others in their network influencing initiatives they considered bringing to their institution
N/A		Silence	A perceived need for greater knowledge of knowledge mobilization processes	Silence	Silence The researcher group theme “a perceived need for greater knowledge of knowledge mobilization processes” pertained to a perceived lack of understanding language and formal processes within KT. This theme did not converge with those raised in other groups
N/A		Context matters	Silence	Silence	Silence The health professional group theme “context matters” related to matters around clinic flow that needed to be considered when engaging in KM activities (e.g., staff coverage, etc.). This theme did not converge with those raised in other groups

### Factor rating task

#### Rating of knowledge mobilization factors

Nearly all partners reported that relationships with leaders and team members, communication quality, and engagement of other partners were very important factors to support successful KM outcomes (Table 7). Size of team or organization, size of external network, and personal knowledge of evidence were the only factors that had ratings of being not very important.

**Table 7:** Ratings of knowledge mobilization factors discussed in interviews

		Health Professional	Researcher	Patient/Caregiver Partner
Factors	Rating	*n* (%)	*n* (%)	*n* (%)
Relationship with leaders and team members	Very Important	10 (100.0)	10 (100.0)	9 (90.0)
	Somewhat Important	0 (0.0)	0 (0.0)	1 (10.0)
	Not Important	0 (0.0)	0 (0.0)	0 (0.0)
Communication quality with team members	Very Important	10 (100.0)	10 (100.0)	10 (100.0)
	Somewhat Important	0 (0.0)	0 (0.0)	0 (0.0)
	Not Important	0 (0.0)	0 (0.0)	0 (0.0)
Size of team or organization	Very Important	3 (30.0)	2 (20.0)	1 (10.0)
	Somewhat Important	5 (50.0)	6 (60.0)	6 (60.0)
	Not Important	2 (20.0)	2 (20.0)	3 (30.0)
Opportunities for networking outside your team or organization	Very Important	4 (40.0)	5 (50.0)	5 (50.0)
	Somewhat Important	6 (60.0)	5 (50.0)	5 (50.0)
	Not Important	0 (0.0)	0 (0.0)	0 (0.0)
Size of team or organization's external network	Very Important	2 (20.0)	2 (20.0)	3 (30.0)
	Somewhat Important	6 (60.0)	7 (70.0)	5 (50.0)
	Not Important	2 (20.0)	1 (10.0)	2 (20.0)
Self-confidence to carry out implementation activity	Very Important	7 (70.0)	7 (70.0)	9 (90.0)
	Somewhat Important	3 (30.0)	3 (30.0)	1 (10.0)
	Not Important	0 (0.0)	0 (0.0)	0 (0.0)
Personal knowledge of evidence to be implemented	Very Important	8 (80.0)	10 (100.0)	5 (50.0)
	Somewhat Important	2 (20.0)	0 (0.0)	4 (40.0)
	Not Important	0 (0.0)	0 (0.0)	1 (10.0)
Personal motivation to participate in implementation activity	Very Important	9 (90.0)	9 (90.0)	7 (70.0)
	Somewhat Important	1 (10.0)	1 (10.0)	3 (30.0)
	Not Important	0 (0.0)	0 (0.0)	0 (0.0)
Implementation plan	Very Important	7 (70.0)	7 (70.0)	8 (80.0)
	Somewhat Important	3 (30.0)	3 (30.0)	2 (20.0)
	Not Important	0 (0.0)	0 (0.0)	0 (0.0)
Engagement of other stakeholders	Very Important	9 (90.0)	10 (100.0)	10 (100.0)
	Somewhat Important	1 (10.0)	0 (0.0)	0 (0.0)
	Not Important	0 (0.0)	0 (0.0)	0 (0.0)

*N* = 30; *n* = 10 in each partner group

#### Ranking of knowledge mobilization factors

Health professionals most commonly reported communication quality, implementation plan, and engagement of other partners within their top three priorities (Table 8). Researchers most frequently endorsed relationships with leaders and team members, communication quality, and engagement of other partners. Patient/caregiver partners most frequently rated communication quality, relationships with leaders and team members, self-confidence to carry out implementation activities, and engagement of other partners within their top three priorities. Communication and engagement were the two most frequently ranked priorities among all partner groups. Team size and network size were not ranked within the top three priorities for any partner groups.

**﻿Table 8:** Knowledge mobilization factor rankings

Factors	Partner Type	1st Priority	2nd Priority	3rd Priority	Cumulative
		*n* (%)	*n* (%)	*n* (%)	*n* (%)
Relationships with leaders and team members	Health Professional	3 (30.0)	2 (20.0)	0 (0.0)	5 (50.0)
	Researcher	3 (30.0)	2 (20.0)	1 (10.0)	6 (60.0)
	Patient/Caregiver	5 (50.0)	1 (10.0)	1 (10.0)	7 (70.0)
Communication quality with team members	Health Professional	4 (40.0)	3 (30.0)	0 (0.0)	7 (70.0)
	Researcher	1 (10.0)	1 (10.0)	3 (30.0)	5 (50.0)
	Patient/Caregiver	1 (10.0)	6 (60.0)	2 (20.0)	9 (90.0)
Size of team or organization	Health Professional	0 (0.0)	0 (0.0)	0 (0.0)	0 (0.0)
	Researcher	0 (0.0)	0 (0.0)	0 (0.0)	0 (0.0)
	Patient/Caregiver	0 (0.0)	0 (0.0)	0 (0.0)	0 (0.0)
Opportunities for networking outside of your team or organization	Health Professional	0 (0.0)	0 (0.0)	1 (10.0)	1 (10.0)
	Researcher	0 (0.0)	0 (0.0)	1 (10.0)	1 (10.0)
	Patient/Caregiver	1 (10.0)	0 (0.0)	1 (10.0)	2 (20.0)
Size of team or organization's external network	Health Professional	0 (0.0)	0 (0.0)	0 (0.0)	0 (0.0)
	Researcher	0 (0.0)	0 (0.0)	0 (0.0)	0 (0.0)
	Patient/Caregiver	0 (0.0)	0 (0.0)	0 (0.0)	0 (0.0)
Self-confidence to carry out implementation activity	Health Professional	0 (0.0)	0 (0.0)	0 (0.0)	0 (0.0)
	Researcher	1 (10.0)	0 (0.0)	0 (0.0)	1 (10.0)
	Patient/Caregiver	2 (20.0)	1 (10.0)	1 (10.0)	4 (40.0)
Personal knowledge of evidence to be implemented	Health Professional	0 (0.0)	1 (10.0)	2 (20.0)	3 (30.0)
	Researcher	1 (10.0)	3 (30.0)	0 (0.0)	4 (40.0)
	Patient/Caregiver	0 (0.0)	0 (0.0)	1 (10.0)	1 (10.0)
Personal motivation to participate in implementation activity	Health Professional	1 (10.0)	0 (0.0)	1 (10.0)	2 (20.0)
	Researcher	0 (0.0)	1 (10.0)	3 (30.0)	4 (40.0)
	Patient/Caregiver	0 (0.0)	0 (0.0)	0 (0.0)	0 (0.0)
Implementation plan	Health Professional	1 (10.0)	1 (10.0)	4 (40.0)	6 (60.0)
	Researcher	2 (20.0)	1 (10.0)	0 (0.0)	3 (30.0)
	Patient/Caregiver	1 (10.0)	2 (20.0)	0 (0.0)	3 (30.0)
Engagement of other stakeholders	Health Professional	1 (10.0)	3 (30.0)	2 (20.0)	6 (60.0)
	Researcher	2 (20.0)	2 (20.0)	2 (20.0)	6 (60.0)
	Patient/Caregiver	0 (0.0)	0 (0.0)	4 (40.0)	4 (40.0)

*N* = 30; *n* = 10 in each partner group

## Discussion

The present study identified contributors that are most impactful in supporting KM activities and partnerships within pediatric pain, from the perspective of health professionals, researchers, and patient/caregiver partners. The findings relate to many of the CFIR constructs [25], supporting the relevance of this framework to understand KM efforts within pediatric pain.

The importance of strong leadership to promote successful KM in pediatric pain was raised by all partners, mapping on to the *opinion leaders* and *implementation leads* constructs of the CFIR Individual domain. The quantitative and qualitative data within all groups converged, describing productive leaders as those who champion initiatives and work collaboratively, all characteristics of effective leadership [46–50]. The roles of team dynamics and diverse expertise were also discussed by all groups, aligning with the Implementation Process construct of *engaging*. Researchers’ quantitative and qualitative data converged on this topic; however, data from the health professionals expanded further, highlighting the importance of communication quality and knowledge of evidence to support KM initiatives. The patients/caregivers group also expanded on team dynamics, highlighting the importance of trust and belongingness, and aligning with the Inner Setting constructs of *culture* and *relational connections*. Taken together, a collaborative leadership style can support constructive team dynamics through increasing information flow, integrating perspectives into decisions, and strengthening communication, all of which increase belongingness and inclusion in KM processes [51–53].

The role of external influences on KM activities was particularly relevant among health professionals and researchers, and the data within both groups converged on this topic. Both described the importance of institutional support to promote the feasibility of engaging in KM initiatives, but with nuances. Health professionals spoke about the role of policy makers and funding in making implementation feasible, contributors relating to the Outer Setting domain constructs of *policies and laws* and *financing*, while researchers spoke to the role of support from academic institutions, within the context of the Inner Setting domain construct of *culture*. These findings are consistent with known barriers to KM, including the need for resources, policy, infrastructure, and support [54–56]. This ultimately speaks to a culture that is incongruent with the broader societal need to increase evidence mobilization [57], especially on issues related to pediatric pain [58]. Also related to external influences, researchers and patient/caregiver partners discussed social contributors, which connects to the Outer Setting construct of *partnerships and connections*. While the quantitative and qualitative data converged among researchers, the patient/caregiver group data diverged, as quantitative ratings showed networks to be less important relative to how they were discussed in the qualitative data. It may be that networks play a supportive role to higher ranked factors, where connections may be necessary to garner resources to support KM activities [59].

Specific concepts within groups also emerged. The importance of the team’s context was raised among health professionals, a finding that relates to the *assessing context* construct of the Implementation Process domain, where contributors such as communication, resources, and time contribute to the functioning of the context [55]. Researchers discussed the importance of knowledge of KM processes, falling within the Individual construct of *capability*, a known barrier to engaging in KM [57].

### Study implications: approaches and strategies to support KM initiatives in pediatric pain

These findings identify what is important to different partners, but also identify shared beliefs about what supports KM activities within children’s pain. Most importantly, these findings highlight rich yet practical opportunities for progressive change in how KM partnerships and outcomes can be supported. Strategies to address these areas can be informed by the Expert Recommendations for Implementing Change (ERIC) taxonomy. The ERIC provides empirically and expert informed implementation strategies to address the areas of importance identified by partners [60, 61]. It is especially useful in work informed by the CFIR, given research that has linked the two (i.e., ERIC strategies can target CFIR determinants) [62]. Informed by the results of the current study, practical opportunities to improve engagement in KM initiatives are discussed, alongside relevant strategies informed by the ERIC taxonomy.

#### Changing the face of leadership

Strong leadership was an evident priority, though it was apparent that researchers and health professionals typically viewed themselves or colleagues as leaders, whereas patient/caregivers did not describe themselves in this role. The lack of representation of diverse perspectives at the leadership level indicates a potential shortcoming in what KM activities are developed in pediatric pain, and thus presents a key opportunity for intervention. Incorporating diverse perspectives (i.e., background and lived experience) in KM activities has been associated with increased partner participation in practice change, reduced conflict among team members, and improved patient outcomes [63]. The ERIC cluster centred around “engaging consumers” highlights strategies such as *involve patients/consumers and family members* and *prepare patients/consumers to be active participants *[60, 61], which can support partners who hold the power to engage patients/caregivers to broaden the scope of who participates in leadership. Including a greater range of partners in leadership can promote equity in implementation when the needs of unique individuals are inherently represented in an initiative [64].

An equally important goal is changing how leaders lead. Given the preference for collaborative leadership, teams may consider adopting a style such as distributed leadership, where power is seen as belonging to the collective team as opposed to a single individual [65]. This style is perceived as being open and approachable, and is shown to increase the flow of information both “up and down” a team’s hierarchy [53]. It also centres around collaboration, where the leader’s role is to enact the vision, values, and goals shared by all partners, while sharing power to make decisions and lead tasks [52, 53]. Distributed leadership can also increase diversity in leadership by sharing power with patient/caregiver partners through greater communication and collaboration [51, 66]. To implement this approach, one can consider the ERIC cluster “develop stakeholder interrelationships,” which includes the strategy *recruit, designate, and train for leadership*. Indeed, the engagement of diverse partners as leaders must be coupled with training opportunities to meet goals around integrating equity into leadership and implementation opportunities [67]. Utilizing these strategies can provide partners with knowledge of effective leadership while simultaneously addressing the need for equity and inclusion at the leadership level.

#### Shifting the culture of how knowledge mobilization activities are valued

The findings of this study also suggest that the value of KM initiatives at an institutional level may influence the types of activities partners have the opportunity or capacity to participate in. Among researchers, the value of KM activities in relation to traditional academic outputs greatly influenced productivity. Inequities in the value of academic outputs can be exacerbated by several factors, including a lack of infrastructure or incentive to engage in KM, heterogenous attitudes toward KM, and challenges developing metrics to compare KM impact and publications [68–70]. If KM activities are to be engaged in more readily, cultural change within institutions around the value of KM, along with infrastructure and resources, are essential. The strategies within the “train and educate stakeholders” ERIC cluster, such as *conduct educational meetings*, can support this goal. Specifically, partners may engage collaboratively with administrators to discuss and define the value of KM, using specific examples of work within the institution to demonstrate impact. Dialogue can be taken a step further through strategies within the “develop stakeholder interrelationships” cluster, such as *involve executive boards*. This strategy supports the involvement of administrators in KM activities, which could include guidelines for engaging in KM activities that align with institutional values. Changing culture is certainly not a short-term task; however, consistent communication regarding the value of KM is critical to create cultural change.

The issue of funding was relevant among health professionals, who reported KM initiatives were most feasible when policy support and funding were available. As such, there is a critical role of funders to dedicate funding to support and protect time for KM activities [71]. Indeed, research has shown that when policy makers prioritize an area of health and increase funding to scale up services, implementation is more easily accomplished [72]. This approach can be supported through the ERIC cluster “utilize financial structures”, where institutions can *fund and contract for the clinical innovation,* subsequently creating opportunities for partners to use strategies such as *access new funding* to make KM initiatives possible. Funding alone is insufficient, however. Strategic policy, practice, and patient-oriented funding calls are necessary to support the identification of priority issues to fund. Models of information exchange can support the exchange of information regarding areas requiring funding support, such as the push model (i.e., knowledge generators sharing evidence), pull model (i.e., evidence requested by knowledge users), and linkage and exchange model (i.e., evidence generation and uptake is promoted in a decision-making context) [73, 74] Utilizing a combination of these approaches would not only ensure that policy- and practice-oriented research is funded, but would also ensure those who can implement the evidence can engage in strategies to receive the necessary support [74]. Overall, strategic decisions are required at the institutional level to ensure the importance of mobilizing knowledge on children’s pain management is funded, valued, and set up for success.

#### Knowledge mobilization on knowledge mobilization

When discussing their participation in KM activities, many researchers described KM and related processes as esoteric, which was attributed to a lack of knowledge, skills, or technical language. This challenge is not unique to pediatric pain researchers, as understanding theories of KM and processes are key needs among researchers wishing to engage in KM [75]. Other researchers’ expressed hesitancy to engage in KM due to ethical concerns and uncertainty about when to share evidence. The ethical imperatives of engaging in KM must be considered prior to engaging [76]; however, KM activities extend well beyond sharing results from a single study and can include evidence syntheses, repositories, briefs, and more [77]. Thus, broadening knowledge of KM, both conceptually and practically, is an area for development among partners.

The ERIC cluster “train and educate stakeholder” addresses this need via strategies such as *conduct ongoing training* and *make training dynamic.* Several training programs have been developed and demonstrated success both in teaching core KM competencies (e.g., theoretical knowledge, skills in developing interventions, synthesis, and research methods, etc.) [78] and increasing confidence in engaging in KM initiatives [79]. Despite the positive outcomes of training initiatives, the uptake among researchers remains low, attributed to time constraints and competing professional priorities [71]. Therefore, low uptake may, in part, be because the onus to engage in training falls primarily on individuals. Institutions should consider taking on the responsibility of encouraging and offering training. This would support partners by providing funding and protected time to participate, something which an academic culture shift could also support. Moreover, integrating training opportunities for graduate students would provide this knowledge and skill at an early stage with the support of mentorship (directly aligned with the ERIC strategy *shadow other mentors*), while also creating the potential for KM to become integrated into trainees research practices throughout their careers [78, 80, 81]. Embedding this training within graduate education is especially important given trainees’ growing interest in integrating KM and partnership approaches to their research [82], and would represent another meaningful shift toward valuing KM activities in academia.

### Strengths and limitations

This study is among the first to explore a combination of perspectives to understand how health professionals, researchers, and patient/caregiver partners differ and converge in their views on what impacts successful KM initiatives within pediatric pain. The convergent mixed-methods design was a significant strength of this study, increasing the validity of the findings through the integration and corroboration of the qualitative and quantitative results. Furthermore, the qualitative data provided rich detail and context to understand why different contributors were important and/or relevant to different groups, adding critical data to inform future KM activities in pediatric pain.

Participants with a range of backgrounds and experiences were purposefully interviewed in this sample, with diversity in ethnicity and country of residence; however, this study would have benefitted from greater representation of racial and cultural identities among participants. This may have influenced the degree to which issues such as social justice and tokenism were raised. This broader lens is critical to support equity-driven KM, increasing the relevance of recommendations to a wider range of partners and generating key considerations for meaningful engagement with diverse individuals [83, 84].

The sample also consisted primarily of cis-gendered women, which may limit the applicability of these findings to KM partners of other sexes and genders. Sex and gender are known to impact participation in decision-making, the expression and reception of evidence-related communication, and ultimately, how KM initiatives are carried out based on the dominant sex and/or gender of the target audience [85, 86]. Future research should examine whether sex and/or gender differences influence partners’ priorities when engaging in KM activities. This may shed additional light on considerations that should be embedded in future guidance on collaboration within multi-partner teams.

## Conclusion

This study demonstrates that collaborative leadership, alongside relationships characterised by shared values and diverse expertise, are critical values within any KM initiative in pediatric pain. While these core contributors are integral to successful KM, accounting for partner-specific differences is essential to ensure everyone can participate in a way that meets their needs and goals. As engagement of diverse partners increases, unique partner needs must be considered, especially to promote equity priorities within implementation. Through strong leadership, innovation, and an openness to change, KM initiatives will become enriched as essential practices in health care to improve pediatric pain management.

### Supplementary Information

**Supplementary Material 1.**

**Supplementary Material 2.**

**Supplementary Material 3.**

## Data Availability

The data for this study has not been shared publicly in order to protect the privacy of participants. Specific requests for the data used in this study are available from the corresponding author on reasonable request and with permission from participants.

## Abbreviations

CAD: Canadian dollar
CFIR: Consolidated Framework for Implementation Research
ERIC: Expert Recommendations for Implementing Change
KM: Knowledge mobilization
SD: Standard deviation
