# Supplementary material for: Understanding the unique and common perspectives of partners engaged in knowledge mobilization activities within pediatric pain management: a mixed methods study
Source: BMC Health Serv Res. 2024 Mar 14;24:337. doi: 10.1186/s12913-024-10782-x (PMC10938714; doi:10.1186/s12913-024-10782-x)
Supplement: Supplementary file 2 — Supplementary Material 2. [file 12913_2024_10782_MOESM2_ESM.pdf]

Understanding the unique and common perspectives of partners engaged in knowledge mobilization activities within pediatric pain: A mixed methods study

MacKenzie et al., 2023

Interview Guide

1. Based on your survey response, you identified primarily as a [partner type]. Can you briefly tell me about that role?

As I mentioned, the purpose of this interview is to understand your experiences with implementation of scientific evidence in children's pain. For the purpose of our discussion, we are defining implementation as structured activities and processes to put interventions or evidence (e.g., recommendations and strategies based on scientific research) into use. In other words, implementation refers to how scientific evidence or research about children's pain is adopted, adapted, used, applied, and shared.

[Check for comprehension]

This definition of implementation also includes dissemination, which we will define as the communication, sharing, and spread of scientific information, through resources like handouts, presentations, infographics, etc.

[Check for comprehension]

2. How does this definition align with your understanding of implementation?

3. Based on these definitions, would you say you have had more experience with dissemination (i.e., evidence sharing), implementation (i.e., application of evidence), or equal experience with both?

4. To begin, tell me about a time you have been involved in implementation of scientific evidence or research about children's pain. How were you involved in the application or sharing of evidence?

5. As a [partner type], what do you think is important to the implementation process or putting interventions or evidence into use within children's pain? In other words, what makes the implementation project *process* successful, in terms of sharing and applying evidence?

6. In what ways do you think team-related factors (for example, the structure of your team, communication or leadership style) impact the implementation process (i.e., sharing and application of evidence)?

7. In what ways do you think elements outside of one's team or organization (e.g., patient needs, peer pressure, external policies/incentives) could impact the implementation process (i.e., sharing and application of evidence)?

8. In what ways do you think an organization's network could impact the implementation process (i.e., sharing and application of evidence)? By network, I am referring to relationships and information sharing with others outside of your team or organization (e.g., other research groups, formal organizations like SKIP/CHC, patient groups, professional or research societies, advocacy groups, etc. They could be local, national, or international)

9. In what ways do you think one's own personal characteristics or qualities) impact the implementation process (i.e., sharing and application of evidence)? By personal qualities, I mean things like self-confidence, knowledge about the evidence, motivation, capacity, etc.

10. What has been your experience with using a plan to guide the implementation process in children's pain? Is this something you have had before when engaged in implementation activities (i.e., sharing and application of evidence)?

11. In your experience with implementation activities in children's pain (i.e., sharing and application of evidence), what types of stakeholders have you worked alongside? This could include health professionals, researchers, patients/caregivers/ family members, and decision makers, among others.

12. Is there anything else that is important to the implementation process within children's pain management from your perspective that you would like to share?
