# Supplementary material for: Understanding the unique and common perspectives of partners engaged in knowledge mobilization activities within pediatric pain management: a mixed methods study
Source: BMC Health Serv Res. 2024 Mar 14;24:337. doi: 10.1186/s12913-024-10782-x (PMC10938714; doi:10.1186/s12913-024-10782-x)
Supplement: Supplementary file 3 — Supplementary Material 3. [file 12913_2024_10782_MOESM3_ESM.pdf]

Understanding the unique and common perspectives of partners engaged in knowledge mobilization activities within pediatric pain: A mixed methods study

MacKenzie et al., 2023

Handout of Key Terms

Defining Stakeholders:
By *stakeholders*, we are referring to individuals or groups engaged or interested (i.e., personally, financially, professionally) in any part of the implementation process. They are individuals or groups who have an interest in, or are affected by, implementation processes.[1] Stakeholders may include:

- Health professionals (e.g., psychologist, physician, nurse, etc.)
- Researchers
- Patients, caregivers, families, lived experience advisor, etc.
- Decision or policy makers
- Health care administrators or leaders
- Community agencies
- Network and/or industry partners
- Research funders or financial/in-kind contributors

The use of the term stakeholders is inclusive of indigenous persons who identify as rights holders. The term stakeholder may not best describe your experience of your past involvement in implementation activities and we invite participants to share their preferred terminology.

Defining Implementation:
By *implementation*, we are referring to structured activities and processes to put interventions or evidence (e.g., recommendations and strategies based on scientific research) into use.[1]

- Implementation refers to how scientific evidence or research about children's pain is adopted, adapted, used, applied, and shared.
- Examples of implementation may include:
    - Participating in clinical practice change
    - Participating in policy change
    - Developing a tailored evidence adoption plan
    - Developing structures to facilitate evidence adoption (e.g., electronic reminder systems)
    - Use of decision aids
    - Work as a local opinion leader
    - Work as a knowledge broker

[1] Canadian Institutes of Health Research. (2012). *Moving into action: We know what practices we want to change, now what? An implementation guide for health care practitioners – Appendices. https://cihr-irsc.gc.ca/e/45670.html#bb*

Our definition of implementation also includes ***dissemination***, which we will define as the active process of communication, sharing, and spread of tailored scientific information.[2]

- Dissemination can happen through making available resources like handouts, presentations, infographics, etc.
- For the interview, when we discuss *implementation*, that will include *dissemination*
- Examples of dissemination may include development of:
    - Plain language summary
    - Policy brief
    - Materials development (e.g., toolkit, pamphlet)
    - Infographics
    - Arts-based knowledge mobilization
    - Clinical practice guideline
    - Stakeholder position paper
    - Engaging with a knowledge broker
    - Developing a stakeholder network

---

[2] Canadian Institutes of Health Research. (2010). *Knowledge translation in health care: Moving from evidence to practice.* https://cihr-irsc.gc.ca/e/40618.html#toc
